# A novel drug discovery strategy: Mechanistic investigation of an enantiomeric antitumor agent targeting dual p53 and NF-κB pathways

**DOI:** 10.18632/oncotarget.2521

**Published:** 2014-10-07

**Authors:** Chunlin Zhuang, Chunquan Sheng, Woo Shik Shin, Yuelin Wu, Jin Li, Jianzhong Yao, Guoqiang Dong, Wen Zhang, Yuk Yin Sham, Zhenyuan Miao, Wannian Zhang

**Affiliations:** ^1^ Department of Medicinal Chemistry, School of Pharmacy, Second Military Medical University, Shanghai, 200433, People's Republic of China; ^2^ Research Center for Marine Drugs, School of Pharmacy, Second Military Medical University, Shanghai, 200433, People's Republic of China; ^3^ Center for Drug Design, Academic Health Center, University of Minnesota, Minneapolis, 55455, Minnesota

**Keywords:** p53-MDM2, NF-κB, antitumor activity, dual inhibitors, enantiomer, molecular dynamics, molecular recognition

## Abstract

The p53 and nuclear factor κB (NF-κB) pathways play crucial roles in human cancer development. Simultaneous targeting of both pathways is an attractive therapeutic strategy against cancer. In this study, we report an antitumor molecule that bears a pyrrolo[3,4-c]pyrazole scaffold and functions as an enantiomeric inhibitor against both the p53-MDM2 interaction and the NF-κB activation. It is a first-in-class enantiomeric inhibitor with dual efficacy for cancer therapy. Synergistic effect was observed *in vitro* and *in vivo*. Docking and molecular dynamics simulation studies further provided insights into the nature of stereoselectivity.

## INTRODUCTION

The p53 tumor suppressor protein is believed to play a crucial role in preventing cancer development by regulating cell cycle and inducing apoptosis [1]. MDM2 protein is a negative regulator of the p53 pathway and its overexpression in cancer cells may lead to inactivation of p53 cellular function. Disruption of the p53-MDM2 interaction is expected to overcome the oncogenic consequences of MDM2 overproduction and restores p53 function [2]. The design and identification of potent p53-MDM2 inhibitors have been avidly pursued in the past decade and several non-peptide small inhibitors such as nutlins [3], benzodiazepines [4], spirooxindoles [5], and piperidinones [6] have been developed as anticancer agents.

The NF-κB is another central mediator of cellular response to external stimuli [7] involved in the control of inflammation, apoptosis and cell proliferation in the human body [8, 9]. Under basal conditions, the NF-κB complex responsible for regulating the transcription of DNA is suppressed by a family of inhibitors called IκBs in the cytoplasm via protein association. Under stimulated conditions, the IκB inhibitory proteins are phosphorylated by IκB kinase (IKK) composed of α, β and γ subunits, resulting in ubiquitination and eventual degradation of the IκBs. The dissociated NF-κB complex subsequently enters the nucleus where it binds to DNA and activates gene expression. Therefore, inhibition of NF-κB signalling by preventing the IKK phosphorylation of IκB proteins has potential therapeutic applications to the treatment of cancer and inflammatory diseases [10].

Recent analysis showed many NF-κB repressors may also function as p53 activators and vice versa [11, 12]. Regulation of the cross-talk between the p53 and the NF-κB pathways would offer a unique therapeutic opportunity for targeting [11]. However, the promiscuous interaction of a small molecule with off-target proteins frequently causes toxicity [13] and adverse effects [14, 15]. Conversely, a single drug target can be therapeutically insufficient, particularly in complex neuropsychiatric conditions, cancers and infectious diseases [16]. Designing compounds with a specific multi-target profile is both complex and challenging [17, 18]. The problem of designing molecules against a multi-target profile involves the parallel optimization of multiple structure-activity relationships within a desired range of physicochemical properties [19].

In our recent study [20], a distinct effort was made to design a p53-MDM2 interaction inhibitors bearing pyrrolidone system through structure-based virtual screening. Subsequently, a novel pyrrolo[3,4-c]pyrazole was rationally designed as a potent inhibitor of the p53-MDM2 protein-protein interaction and the NF-κB pathway. A dual inhibitor is preferred over the combined use of two single-target p53-MDM2 and NF-κB inhibitors because it eliminates the need to optimize individual inhibitor doses for efficacy and the potential complications of drug-drug interactions. In this study, racemic pyrrolo[3,4-c]pyrazole was demonstrated as a first-in-class small molecule being used simultaneously as a p53-MDM2 and NF-κB inhibitor. The p53-MDM2 inhibition was confirmed by cell-free biochemical assay and cell-based Western blot. Meanwhile, the racemic compound was also shown to suppress NF-κB nuclear translocation by inhibiting the IκB kinases of NF-κB pathway [21].

**Figure 1:**
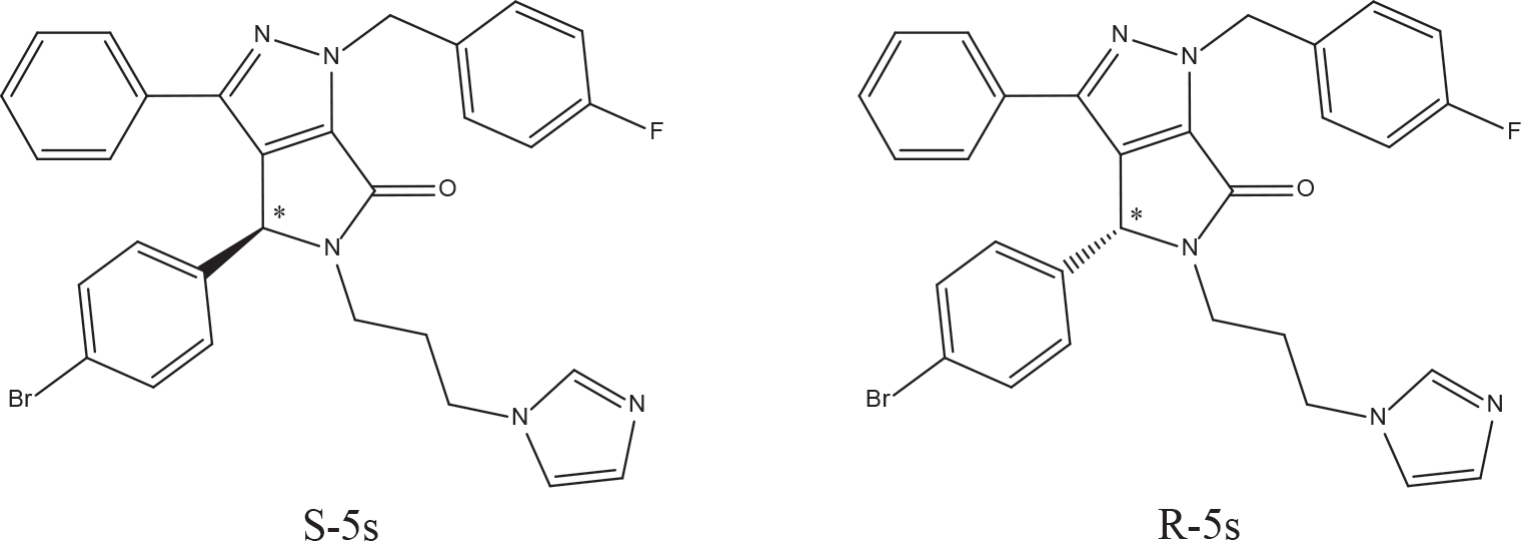
Chemical structures of enantiomeric compound R-5s and S-5s The asterisk indicates the location of the sole chiral center.

The central goal of this study is to determine whether the two enantiomers (see Supplementary information for NMR spectra and chiral separation, Figure S1) of the newly designed racemic compound [21] of 5-(3-(1*H*-imidazol-1-yl)propyl)-4-(4-bromo-phenyl)-1-(4-fluorobenzyl)-3-phenyl-4,5-dihydropyrrolo[3,4-c]pyrazol-6(1*H*)-one (Figure 1), could respectively target these two different pathways with promising selectivity. This hypothesis was supported by a series of biological assays and computational studies.

## RESULTS

**Figure 2:**
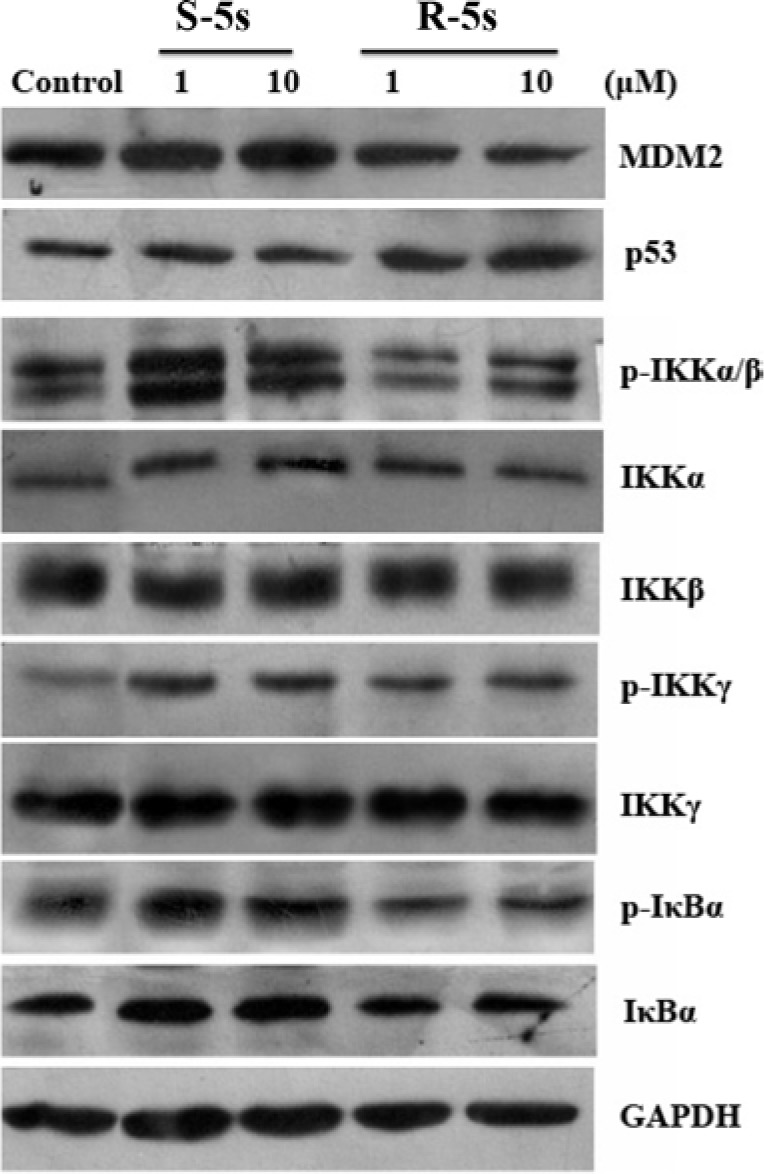
Cellular activity of the two enantiomers on the p53 and NF-κB pathways as detected by Western blotting assay (A549 cells, 4 h treatment)

**Figure 3:**
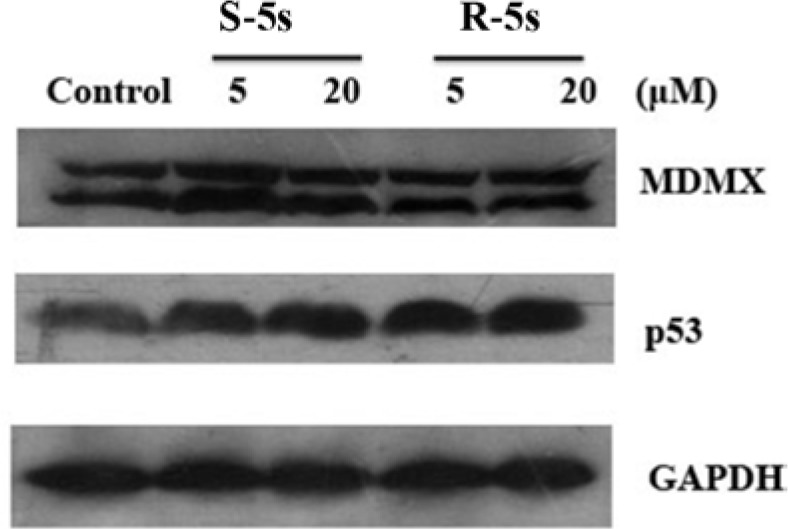
Cellular activity of the two enantiomers on p53 and MDMX as detected by Western blotting assay (MCF-7 cells, MDMX overexpression, 4 h treatment)

To quantify the level of p53-MDM2 inhibition by the enantiomers of compound 5s, Western blot analysis was carried out to analyze p53 and MDM2 protein levels in A549 cells. Interestingly, compound R-5s increased the p53 protein level in a dose-dependent manner and inhibited MDM2 expression after 4h treatment (Figure 2), while no significant change was observed in compound S-5s treated cells. In MCF-7 cells, similar results were observed for p53, while no significant change was observed for MDMX, a homolog oncoprotein of MDM2 (Figure 3). These findings were consistent with the biochemical assay of MDMX (K_i_> 100 μM, data not shown).

To determine differences in their bioactivity on the NF-κB pathway, the relative cytoplasmic levels of IκBα and IKKs from the same cell lysates were examined by Western blot assay (Figure 2). Contrary to the p53 pathway, both enantiomers inhibited phosphorylated IκBα. Compound S-5s exhibited both higher activity and concentration-dependency as compared with R-5s. For IκB kinases, incubation of the cells with compound S-5s only for 4h markedly activated the phosphorylation of IKKβ only or both IKKβ and IKKα and phosphorylated IKKγ in a dose-dependent manner, with no obvious changes in total IKK proteins. Similar behaviour for IκBα with no differences was observed when treated with the other enantiomer, indicating that the compound S-5s of pyrrolo[3,4-c]pyrazole compound suppressed NF-κB activation through inhibition of IκBα phosphorylation mediated by the IKKs phosphorylation. The similar but much weaker effect of the other R-5s was probably induced by the p53 pathway rather than by direct inhibition of the NF-κB pathway.

The *in vitro* antiproliferative activity shown in Table 1 demonstrated that the two enantiomers had a distinct synergistic effect against various cell types. The IC_50_ values of the two enantiomers were 27.4 and 21.2 μM against A549 cells, respectively, while the racemic exhibited a non-additive synergistic enhancement of 5.82 μM. The similar effect was observed in both H1299 and U2 OS cells, but not obviously in Saos-2 cells. Western blot study showed that the S-5s could inhibit NF-κB activation only, while the R-5s could inhibit both p53-MDM2 interaction and induce the inhibition of NF-κB activation with the *in vitro* antiproliferative result indicating poor selectivity over cancer cell lines (H1299 and Saos-2) with deleted p53.

**Table 1:** *In vitro* antiproliferative data of compound 5s

Compound	MTT-IC_50_ (μM)			
	A549	H1299	U2 OS	Saos−2
Racemic-5s	5.82 ± 0.04	2.39 ± 0.19	2.09 ± 0.02	5.08 ± 0.55
S-5s	27.4 ± 1.23	4.02 ± 0.23	9.2 ± 1.42	13.5 ± 1.04
R-5s	21.2 ± 1.52	5.07 ± 0.14	4.0 ± 0.30	4.2 ± 0.16
Nutlin-3	20.79 ± 1.43	16.34 ± 5.59	12.76 ± 2.42	4.14 ± 0.10

All the experiments were duplicated. Data are presented as the mean ± SD.

**Figure 4:**
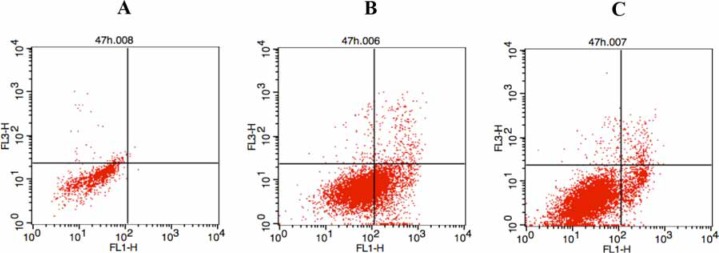
Two enantiomers-induced cell apoptosis **(A)** A549 cells were treated with DMSO as control; **(B)** 10 μM of compound S-5s for 47 h; (C) 10 μM of compound R-5s for 47 h.

Considering that both the p53 and NF-κB were involved in the control of apoptosis [1, 22], the effect of the two enantiomers on the induction of A549 cell apoptosis was evaluated by fluorescence-activated cell sorting (Figure 4). After 47 h treatment at the concentration of 10 μM, the percentage of apoptotic cells for the two enantiomers was about 4.18% and 2.92%, respectively, while no significant apoptotic effect was demonstrated in the control group.

**Figure 5:**
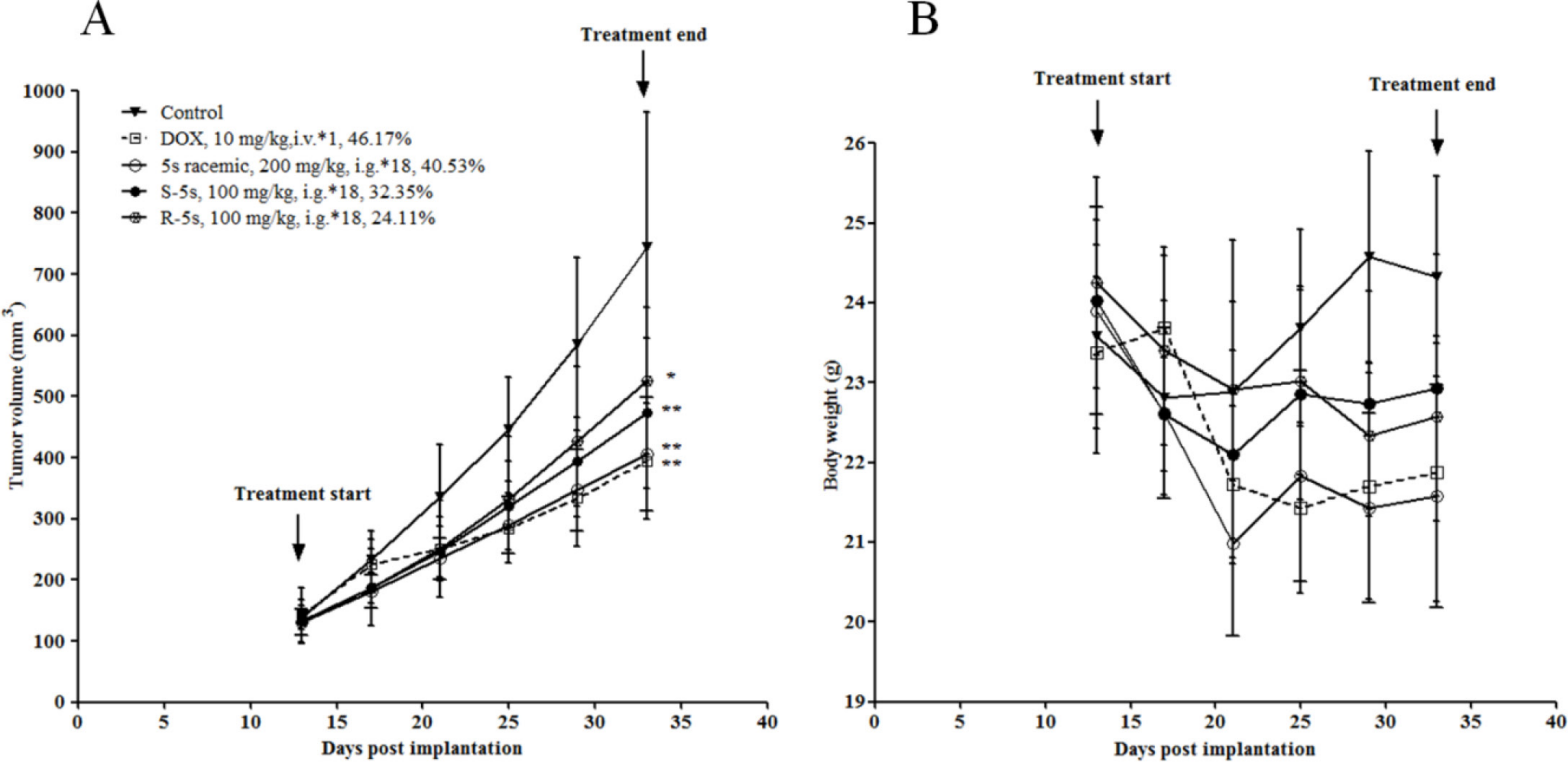
Antitumor activity of compound 5s (200 mg/kg, administered intragastrically, 18 days), and compounds S-5s and R-5s (100 mg/kg, administered intragastrically, 18 days) against A549 xenografts in nude mice **(A)** tumor volume; **(B)** animal body weight. Data are presented as the mean ± SEM; n = 6 nude mice per group: (*) *p*< 0.05, (**) *p*< 0.01, versus control group (n = 10 nude mice), determined with Student's t test.

To investigate the *in vivo* effect of the two enantiomers on tumor growtha, an A549 xenograft mouse model was prepared to evaluate compound 5s racemic at the dose of 200 mg/kg, while the dose of 100 mg/kg was used for the two single enantiomers. As depicted in Figure 5, intragastrical (i.g.) administration of these compounds for 18 days significantly inhibited tumor growth (*p* < 0.05). The tumor volume was inhibited by 40.53% (racemic), 32.35% (S-5s) and 24.11% (R-5s), respectively. The two enantiomers were found to be well tolerated during the test and showed no significant loss of body weight as compared with the reference drug doxorubicin (DOX) (Figure 5). The above results also indicate that the two enantiomers had some synergistic effect both *in vitro* and *in vivo*.

**Figure 6:**
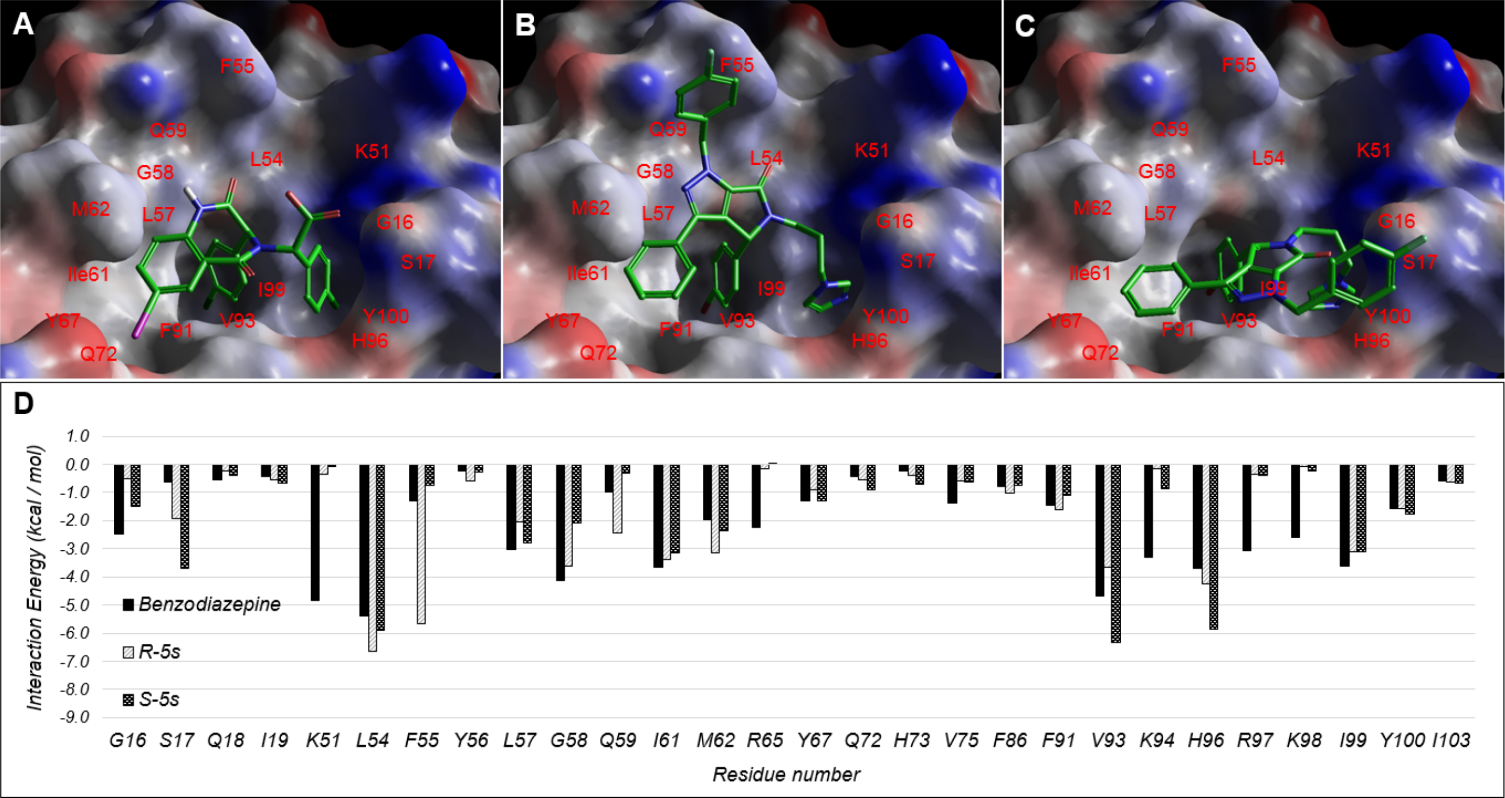
Observed Binding mode of (A) benzodiazepine and docking poses of (B) R-5s and (C) S-5s within the p53-binding site of MDM2. (D) Per residue interaction energy diagram for benzodiazepine (Blue), R-5s (orange) and S-5s (green) within 12Å of MDM2 ligand binding site. Only interactions greater than absolute 1 kcal/mol are shown

Docking studies were performed to examine the potential mode of binding for R-5s and S-5s in MDM2 (Figure 6). The result showed both enantiomers bind similarly to the crystallographically observed benzodiazepine, indicating that insertion of its aromatic substituents into the three primary sub-pockets of MDM2 was crucial for molecular recognition. The interactions involved were the two phenyl rings with the two hydrophobic I61 and V93 sub-pockets and the diazole ring binding to the H96 sub-pocket via π-π interaction. The primary scaffold of the pyrrolo[3,4-c]pyrazole group in S-5s occupied the same L54 and G58 region as benzodiazepine with a single hydrogen bond formed between its lactam oxygen atom and L54. This binding conformation also allowed the benzyl group of R-5s to bind outside the binding hotspot region of MDM2 and formed an additional π-π interaction with F55 which was neither possible in benzodiazepine nor S-5s. The overall docking score for R-5s and S-5s was -7.6 and -6.7, respectively *vs*. -7.6 of benzodiazepine which could corroborate our observed higher bioactivity of R-5s over S-5s against the p53-MDM2 pathway.

For IKKβ, the original solved X-ray structure consists of XNM (4-((4-(4-(chlorophenyl) pyrimidin-2-yl) amino) phenyl) (4-(2-hydroxyethyl) piperazin-1-yl) methanone) that binds to a series of connected hydrophobic pockets consisting of L21, V29, A42, I65, Y98, and G102 with hydrogen bonding to K106 (Figure S2). The observed binding mode for both R-5s and S-5s showed binding poses that recapitulate the interactions of XNM by binding to the same sites with its primary scaffold while extending its halo substituted phenyl ring into the V29 and I26 sub-pocket, forming a salt bridge between its negatively charged diazole group with K105. The difference in the mode of binding between S-5s and R-5s was the placement of its primary scaffold and the subsequent placement of all four of its aromatic rings into various sub-pockets of IKKβ. For S-5s, the placement of its core scaffold near E149 resulted in a favourable non-bond interaction of nearly −5kcal/mol which was not found in XNM and R-5s. The overall docking score for R-5s and S-5s was -4.1 and -5.4, respectively *vs*. -7.6 of XNM. The observed docking score was relatively low that suggests both enantiomers could be a relatively weak inhibitor as compared to XNM with S-5s as the more potent of the two for potentiating the synergistic bioactivities of compound 5s.

Molecular dynamics (MD) simulations were carried out to further establish the dynamical nature of the essential interactions involved in molecular recognition (Figure 8). The 100ns simulations for the original X-ray structures of MDM2 with benzodiazepine exhibited an average C_α_RMSD of 1.0Å with a standard deviation with 0.1Å, indicating the existence of a tightly bound complex that did not allow significant conformational changes after complexation. For IKKβ with XNM, the average C_α_RMSD for the ligand binding IKKβ KD domain was 2.9Å (Figure S3) with a standard deviation with 0.5Å, indicating a more conformation flexible target. For the two enantiomeric complexes, the simulations showed that the predicted modes of binding were maintained with a relatively slight increase in average C_α_RMSD for MDM2 with R-5s and a decrease in average C_α_RMSD for IKKβ with S-5s at 1.5Å and 2.4Å, respectively. The ability to lower the conformational flexibility of IKKβ KD domain suggested the potentiality of S-5s as an allosteric protein-protein interaction inhibitor (PPIi) for modulating IKKβ's affinity for the NF-κB complex.

Finally, the average nearest interatomic distances between the inhibitors and the essential residues with the ligand binding site identified from the docking study are shown in Table 2. For MDM2, all but two of the distances between MDM2 and either benzodiazepine or R-5s were conserved with less than 1.0Å differences. R-5s with an additional methylene spacer between its core scaffold and the diazole group allowed it to penetrate deeper into the His96 sub-pocket as compared with benzodiazepine. Also as shown in the docking study and the per energy interaction diagram, R-5s could extend its benzyl group to Phe55 to form an additional π-π interaction with an average nearest interatomic distance of 4.6Å. For IKKβ, similar conserved distances were observed including K106 with the exception of E149. The placement of S-5s core scaffold near E149 at the average nearest interatomic distance of 3.4Å explained why the observed favourable non-bond interaction of −5kcal/mol was distinctively unique from R-5s and XNM (Figure S4).

**Table 2:** Average nearest interatomic distances between inhibitors and binding site residues

Residues	MDM2			IKKβ	
	benzodiazepine	R-5s	Residues	XNM	S-5s
Leu54	4.2	4.5	Leu21	4.2	3.8
Phe55	6.3	4.6	Met96	5.3	4.6
Gly58	4.2	4.3	Tyr98	4.7	4.9
Gln59	5.5	4.4	Asp103	4.5	3.8
Ile61	3.8	4.0	Lys106	4.3	4.0
Val93	4.0	3.8	Glu149	7.6	3.4
His96	5.5	4.6	Ile165	3.6	4.0
Ile99	3.8	3.8			

The essential residues within the ligand binding site were identified from the docking study. The distances are averaged over the course of the simulation and are shown in Å.

## DISCUSSION

In the present study, we reported a first-in-class enantiomeric inhibitor with dual efficacy for cancer therapy. Our previous report demonstrated the chiral center played a crucial role in the MDM2 binding activity [21]. For the two enantiomers of compound 5s obtained by chiral separation, we found only R-5s could inhibit p53-MDM2 interaction and release p53 to suppress tumor proliferation. Molecular modelling further corroborates with our biochemical studies. Like most p53-MDM2 inhibitors, R-5s could mimic the three residues of the p53 and insert into three sub-pockets of MDM2 with a much higher docking Gscore of −7.652 (Figure 6B). However, S-5s interacted with MDM2 by only filling its Phe19 and Trp23 sub-pockets with its two aromatic substituents (Figure 6C).

**Figure 7:**
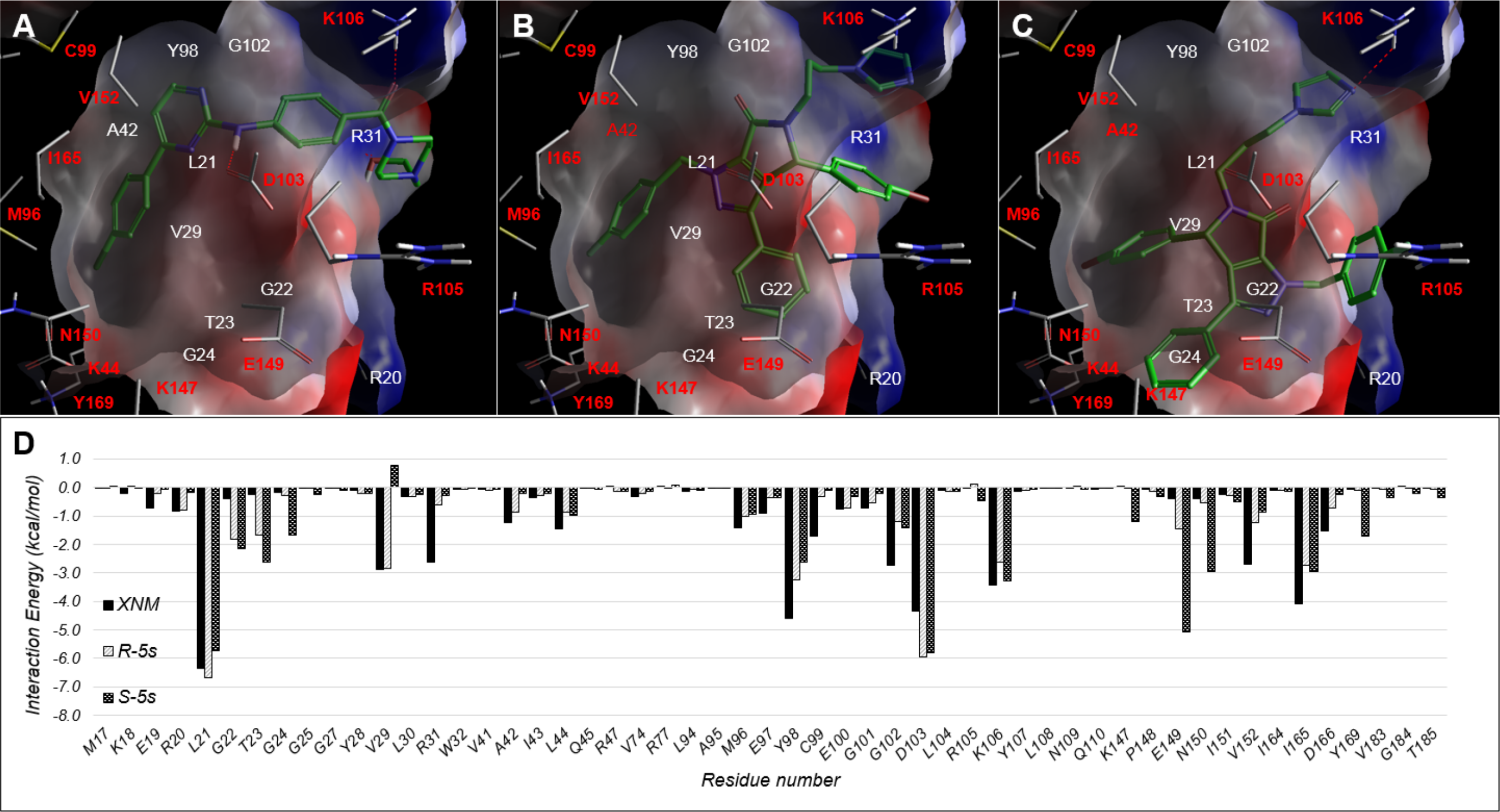
Observed Binding mode of (A) XNM and docking poses of (B) R-5s and (C) S-5s within the ligand binding IKKβ KD domain. (D) Per residue interaction energy diagram for XNM (Blue), R-5s (orange) and S-5s (green) within 12Å of MDM2 ligand binding site. Only interactions greater than absolute 1 kcal/mol are shown

Interestingly, the inactive p53-MDM2 inhibitor S-5s demonstrated promising *in vitro* antiproliferative activity as compared with active enantiomer R-5s. On the other hand, racemic compound 5s was illustrated to efficiently suppress NF-κB activation by inhibiting NF-κB protein translocation to the nucleus [21]. In this study, we demonstrated that S-5s could inhibit IκBα phosphorylation and markedly activate the phosphorylation of IKKβ only or both IKKβ and IKKα and phosphorylated IKKγ in a dose-dependent manner only for 4 h. Therefore, S-5s could inhibit NF-κB signalling via promoting the IKK phosphorylation and preventing the phosphorylation of IκB proteins. In addition, most NF-κB repressors have been found to be p53 activators and vice versa [11, 12]. The Western blotting resultshowed that R-5s could inhibit the p53-MDM2 binding in the cells and subsequently induce the NF-κB inhibition (Figure 2). Computational study predicted that the S-5s had a better docking Gscore (−5.441) with IKKβ protein than R-5s (Gscore = −4.143) (Figure 7).

**Figure 8:**
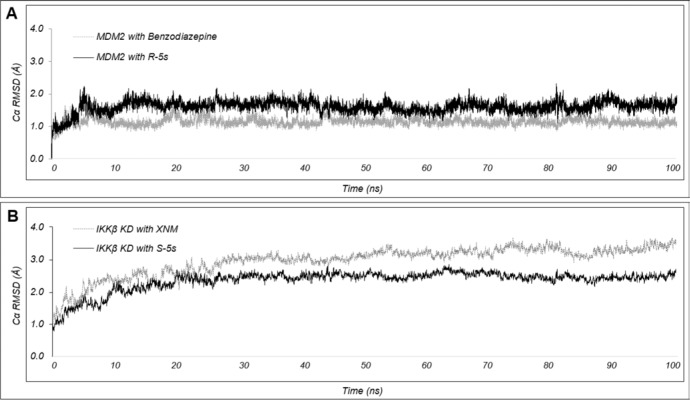
Plot of C_α_RMSD for the inhibitor bound (A) MDM2 and (B) IKKβ complexes over the course of 100ns MD simulation Simulations with the enantiomeric inhibitors (R-5s and S-5s) are shown in black while the structural solved inhibitors (benzodiazepine and XNM) are shown in grey

It was hypothesized that drug combination acting more than one target could enhance normal tumor sensitivity and increase therapeutic indices. However, this hypothesis is being challenged clinically because it is no easier to achieve a tolerable drug level. It was found in this study that two enantiomers of one compound could lower the risk of drug combination and targets two different pathways simultaneously. It is also encouraging to see that the two enantiomers had a synergistic effect in three cell lines except Saos-2. For the *in vivo* efficacy, the enantiomers reduced the tumor volume by 32.35% for S-5s and 24.11% for R-5s *vs*. 40.53% for the racemic compound 5s (*p* < 0.01), which is not significantly lower than 46.17% for DOX (*p* < 0.01). The above result indicated an apparent synergistic *in vivo* effect. In conclusion, we have characterized the mechanism of action of the two enantiomers of pyrrolo[3,4-c]pyrazole compound 5s. R-5s was found to be potent toward MDM2 and might induce NF-κB inhibition. S-5s could selectively suppress NF-κB activation by inhibiting IκBα phosphorylation and the cytoplasmic level elevation of phosphorylated IKKs. The enantiomers had a synergistic effect both *in vitro* and *in vivo*. The results of computational analysis further corroborate with the above biological study. The present study has further demonstrated that MD simulations are a powerful tool to predict the atomistic behaviour of tightly binding inhibitors. The obtained interatomic distance measurements indicate that the final binding poses of the two enantiomers are very stable as compare with initial molecular docking model. Further structural optimization to identify the inhibitors of these two pathways with much higher selectivity is ongoing in our laboratory.

## METHODS

### *In vitro* antiproliferative assay

The cellular growth inhibitory activity was determined using two human osteosarcoma cell lines [U-2 OS and Saos-2] and two human lung cancer cell lines [A549 and NCI-H1299]. An amount of 5-6 × 10^4^ cells per well was transferred to 96-well plates. After culturing for 24 h, the test compounds were added to triplicate wells at serial diluted concentrations and 0.1% DMSO for control. After 72 h of incubation, 20 μL of MTT (3-[4,5-dimethylthiazol-2-yl]-2, 5-diphenyltetrazolium bromide) solution (5 mg/mL) was added to each well, and after the sample was shaken for 1 min, the plate was incubated further for 4 h at 37 °C. The compounds were dissolved in 100 μL of DMSO. The absorbance (OD) was quantitated with the microplates using Biotek Synergy H2 at 570 nm. Wells containing no drugs were used as blanks. The concentration of the compounds that inhibited cell growth by 50% (IC_50_) was calculated. Nutlin-3 was used as a reference compound.

### Western blotting assay

A549 or MCF-7 cancer cells with wild-type p53 were grown in the recommended medium supplemented with 10% FBS (Invitrogen) in a humidified environment with 5% CO_2_. After 4 h treatment with various concentrations of the compounds, cells were lysed and the protein extract was denatured and run on 5% Trisglycine polyacrylamide gels (Invitrogen). Gels were electroblotted onto nitrocellulose membranes, and Western detection was carried out using 5% milk buffer (5% nonfat dry milk in TBS/0.1% Tween-20) throughout. Proteins were detected by ECL chemiluminescence reagents (Pierce, #32209) using antibodies specific for human p53 (Calbiochem, #OP43T), MDM2 (Millipore, #07−575), MDMX (abcam, #ab16058), NF-κB p65 (D14E12) Rabbit mAb (CST, #8242S), NF-κB (p105/p50) (NFKB1) antibody (Epitomic, #1559-1), IKKα (abcam, #1615-1), IKKβ (IKBKB) antibody (abcam, #3902-1), IKKγ (DA10-12) Mouse mAb (CST, #2695S), Phospho-IKKα/β (Ser176/180) (16A6) Rabbit mAb (CST, #2697S), Phospho-IKKγ (Ser376) Antibody (CST, #2689S), Phospho-IκBα (Ser32) (14D4) Rabbit mAb (CST, #2859S), IκBα (44D4) Rabbit mAb (CST, #4812S), GAPDH antibody (multiscience, #mab5465), and Histone 3 (D1H2) XP® Rabbit mAb (CST, #4499P).

### Apoptosis analysis by flow cytometry

A549 cells (5 × 10^5^ cells/mL) were seeded in six-well plates and treated with the compounds at the concentration of 10 μM for 47 h. The cells were then harvested by trypsinization and washed twice with cold PBS. After centrifugation and removal of the supernatants, cells were resuspended in 400 μL 1X binding buffer, which was then added to 5 μL annexin V-FITC and incubated at room temperature for 15 min. After addition of 10 μL of PI, the cells were incubated at room temperature for another 15 min in the dark. The stained cells were analyzed with a flow cytometer (BD Accuri C6).

### *In vivo* antitumor study

The *in vivo* antitumor activity of 5s and its enantiomers was evaluated using DOX as the reference drug. BALB/C nude male mice (Certificate SCXK2003-0003, weighing 18−20 g) were obtained from Shanghai Experimental Animal Center of Chinese Academy of Sciences. A549 cancer cell suspension was implanted subcutaneously into the right axilla region of the mice. Treatment was initiated when the implanted tumor grew to a volume of about 120 mm^3^ in after 13 days. The animals were randomized into appropriate groups (6 animals per treatment group and 10 animals for the control group) and administered by gavage once daily for 18 consecutive days from day 13 after implantation of the cells. Tumor volumes (TV) were monitored by caliper measurement of the length and width and then calculated using the formula: TV = 1/2 × a × b^2^, where a is the tumor length and b is the width. Tumor volumes and bodyweights were monitored every 4 days throughout the course of treatment. Mice were sacrificed on day 33 after cell implantation, and tumors were removed and recorded for analysis. Tumor volume inhibition was determined by the formula of 1-RTV_tumor_/RTV_control_× 100%, where RTV is the relative tumor volume to that of day 13. DOX (10 mg/kg, Pfizer Italia S.r.l) was administered intravenously once on day 13 after implantation.

### Molecular docking

The standard protocol for the docking study used for distinguishing the different potential mode of binding among enantiomers has been described in detailed elsewhere [23]. Briefly, All docking studies were carried out using the Schrodinger modeling suite package [24]. The crystallographic structures of the targeted MDM2 (PDB code: 1T4E) [4] and IKKβ (PDB code: 3RZF) [25] with bound inhibitors were used as the starting point for examining the potential mode of binding for the two enantiomers of compound 5s. All crystallographic waters and ions were removed prior to the addition of the missing hydrogen atoms according to the ionizable states at physiological pH. The prepared protein structures were energy minimized using OPLS-AA 2005 force field [26] to optimize all hydrogen-bonding networks. Both enantiomers of compound 5s were built and docked into the prepared protein structure without restraints using the Standard Precision protocol of Schrodinger's Glide v5.6. To identify the key amino residues involved in the molecular recognition that distinguish between the two enantiomers, the non-bond per residue interaction energies between each docked ligand to residues within 12Å of the targeted binding site were evaluated with a constant dielectric constant of 4.

### Molecular dynamics simulation

MD simulations were carried out for MDM2 and IKKβ in complex with the structurally solved inhibitors and with the more potent of the two enantiomers. Each system was solvated in a cubic box with explicit TIP3P water [27] and counter ions consisting of a 10Å solvent buffer region from the edge of the complex. The long range electrostatic interactions were evaluated by the Particle-Mesh Ewald method under the periodic boundary condition. 100 ns simulation was carried out for each docked model using DESMOND [28] with OPLS-AA 2005 force field under the isobaric isothermal (NPT) condition at 300K and 1 atm. The stability of the simulation was assessed by monitoring the C_α_RMSD with respect to the minimized starting structure. For IKKβ consisting of KD, ULD and SDD domains, the C_α_RMSD was evaluated for the ligand binding KD domain.

## SUPPLEMENTARY FIGURES AND TABLES


