# Supplementary material for: A novel drug discovery strategy: Mechanistic investigation of an enantiomeric antitumor agent targeting dual p53 and NF-κB pathways
Source: Oncotarget. 2014 Oct 7;5(21):10830–9. doi: 10.18632/oncotarget.2521 (PMC4279413; doi:10.18632/oncotarget.2521)
Supplement: Supplementary file 1 [file oncotarget-05-10830-s001.pdf]

SUPPLEMENTARY METHODS, TABLE AND FIGURES

Characterization for compound 5s and its enantiomers

(±)-5-(3-(1*H*-imidazol-1-yl)propyl)-4-(4-bromophenyl)-1-(4-fluorobenzyl)-3-phenyl-4,5-dihydropyrrolo[3,4-*c*]pyrazol-6(1*H*)-one (5s). Yield 64.3%, yellow solid, Mp: 113-115 ºC. ^{1}H NMR (300 MHz, DMSO-d_6) δ: 7.62 (s, 1H), 7.47-7.56 (m, 6H), 7.20-7.31 (m, 7H), 7.15 (s, 1H), 6.88 (s, 1H), 6.02 (s, 1H), 5.54 (s, 2H), 3.97 (m, 2H), 3.60 (m, 1H), 2.67 (m, 1H), 1.90 (m, 2H). ^{13}C NMR (75 MHz, DMSO-d_6) δ: 164.26, 161.03, 158.68, 143.94, 141.12, 138.06, 135.86, 133.72, 132.93, 131.99, 131.06, 130.91, 130.73, 129.42, 129.06, 128.98, 126.58, 122.79, 120.04, 116.55, 116.27, 58.70, 53.37, 44.36, 38.45, 30.18. ESI-MS (m/z): 570.30 [M+H].

(+)-5-(3-(1H-imidazol-1-yl)propyl)-4-(4-bromophenyl)-1-(4-fluorobenzyl)-3-phenyl-4,5-dihydropyrrolo[3,4-c]pyrazol-6(1H)-one (5s-1). Mp: 82-83 ºC. ^{1}H NMR (600 MHz, DMSO-d_6) δ: 7.61 (s, 1H), 7.53 (d, 2H, J = 8.4 Hz), 7.48-7.50 (m, 4H), 7.21-7.30 (m, 7H), 7.15 (s, 1H), 6.88 (s, 1H), 6.02 (s, 1H), 5.55 (d, 2H, J = 4.8 Hz), 3.96 (m, 2H), 3.60 (m, 1H), 2.67 (m, 1H), 1.90 (m, 2H). ESI-MS (m/z): 570.51 [M+H]. $[\alpha]_D$ = +94.9° (c = 1.26 in CH_3OH).

(-)-5-(3-(1H-imidazol-1-yl)propyl)-4-(4-bromophenyl)-1-(4-fluorobenzyl)-3-phenyl-4,5-dihydropyrrolo[3,4-c]pyrazol-6(1H)-one (5s-2). Mp: 81-82 ºC. ^{1}H NMR (600 MHz, DMSO-d_6) δ: 7.60 (s, 1H), 7.53 (d, 2H, J = 9.0 Hz), 7.48-7.50 (m, 4H), 7.21-7.30 (m, 7H), 7.14 (s, 1H), 6.87 (s, 1H), 6.01 (s, 1H), 5.54 (d, 2H, J = 4.8 Hz), 3.96 (m, 2H), 3.60 (m, 1H), 2.67 (m, 1H), 1.90 (m, 2H). ESI-MS (m/z): 570.46 [M+H]. $[\alpha]_D$ = -85.6° (c = 1.24 in CH_3OH).

Compound (+)-5s-1

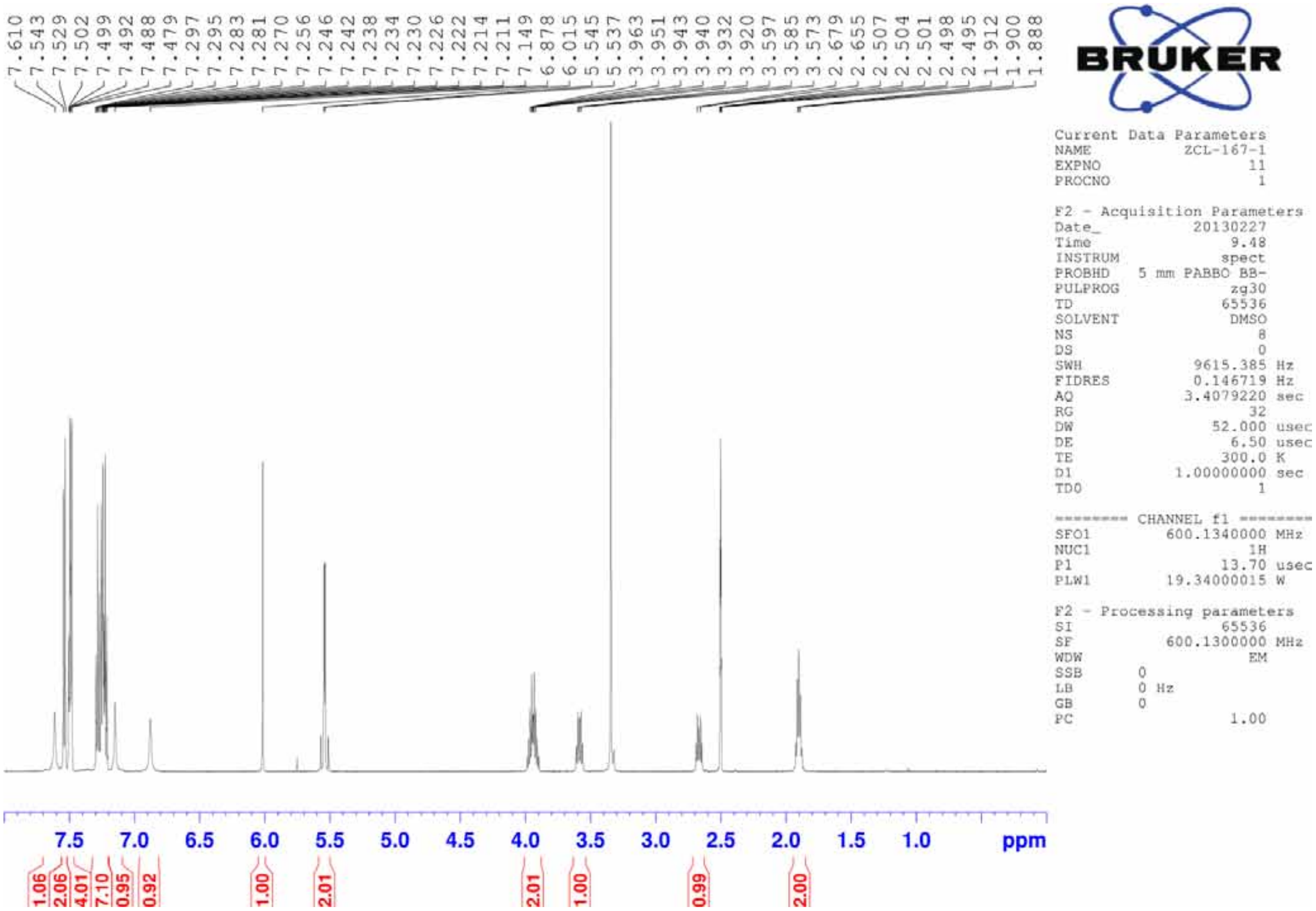

Compound (-)-5s-2

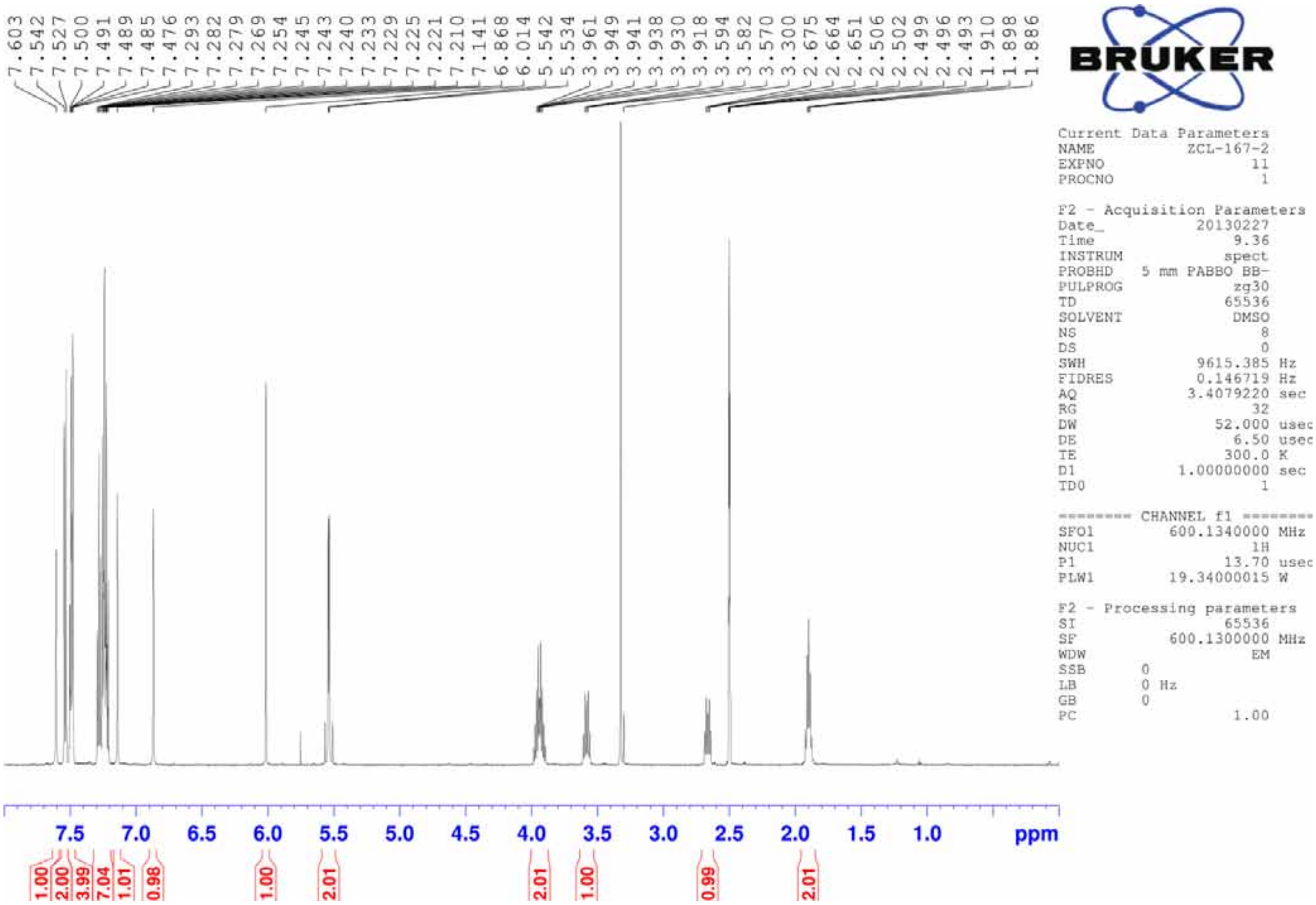

Chiral separation for compound 5s

As depicted in Table S1 and Figure S1, the racemic compound 5s was separated using OJ-H chiral column. The retention time for these two enantiomers is 3.3 and 4.3 min, respectively. The purity is both over 95.0%.

Supplementary Table S1. Chiral separation method

**Column**	**OJ-H**
**Column Size**	0.46 cm I.D. *25 cm L
**Mobile phase**	SF CO_2/MeOH (0.1% DEA) = 60/40
**Flow rate**	2.5 mL/min
**Wavelength**	UV 220 nm
**Temperature**	40 ºC

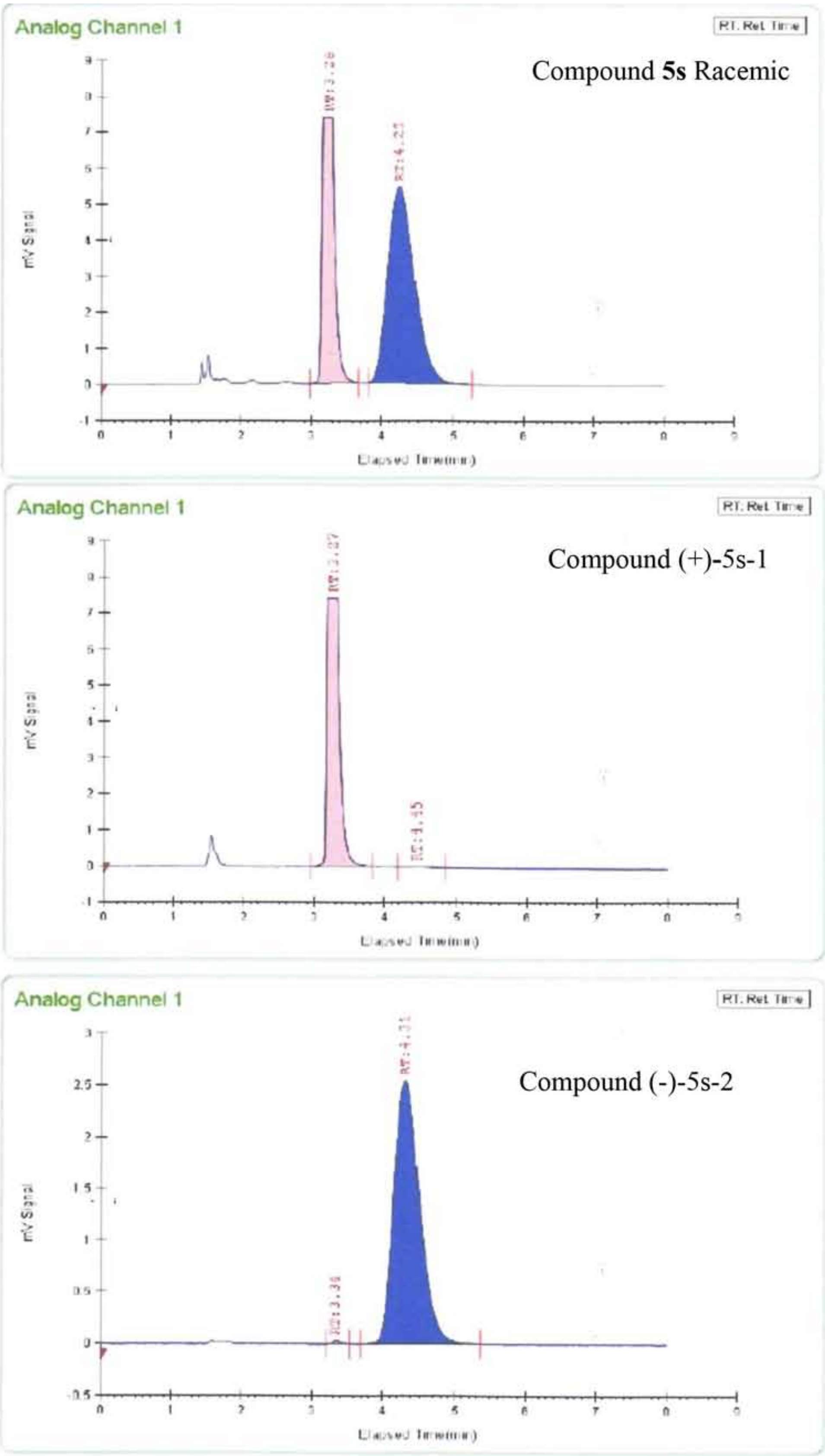

Supplementary Figure S1: Chiral separation spectra of the racemic compound 5s and its enantiomers.

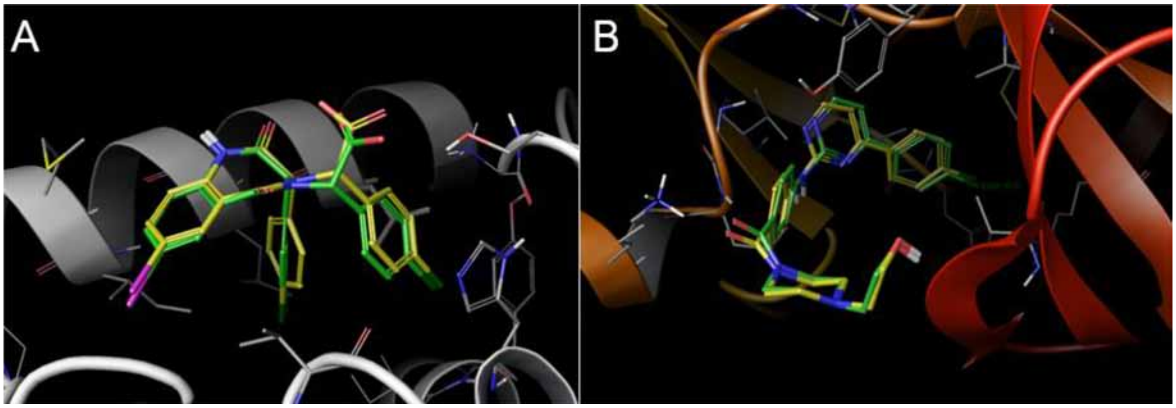

Supplementary Figure S2: Successful re-docking of (A) benzodiazopine and (B) XNM back into its original X-ray structures by the Standard Precision protocol of Schrodinger's Glide v5.6 used in the docking study.

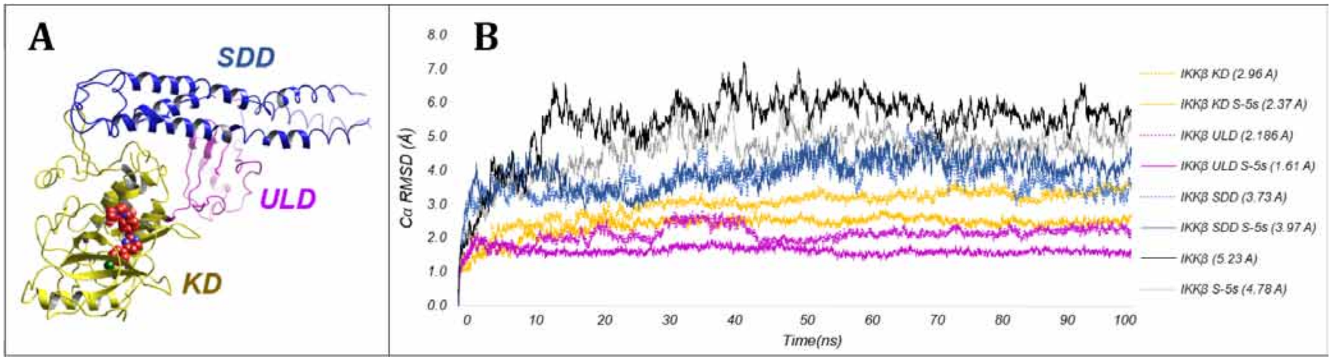

Supplementary Figure S3: (A) Ribbon representation of IKKβ with its highlighted KD (yellow), SDD (blue and ULD (magenta) domains. Inhibitor XNM is shown in red space-filling model to highlight the location of the ligand binding site in KD. (B) Plot of $C_α$RMSD for the KD, ULD and SDD domains of IKKβ in complex to XNM and S-5s over the course of 100ns MD simulation.

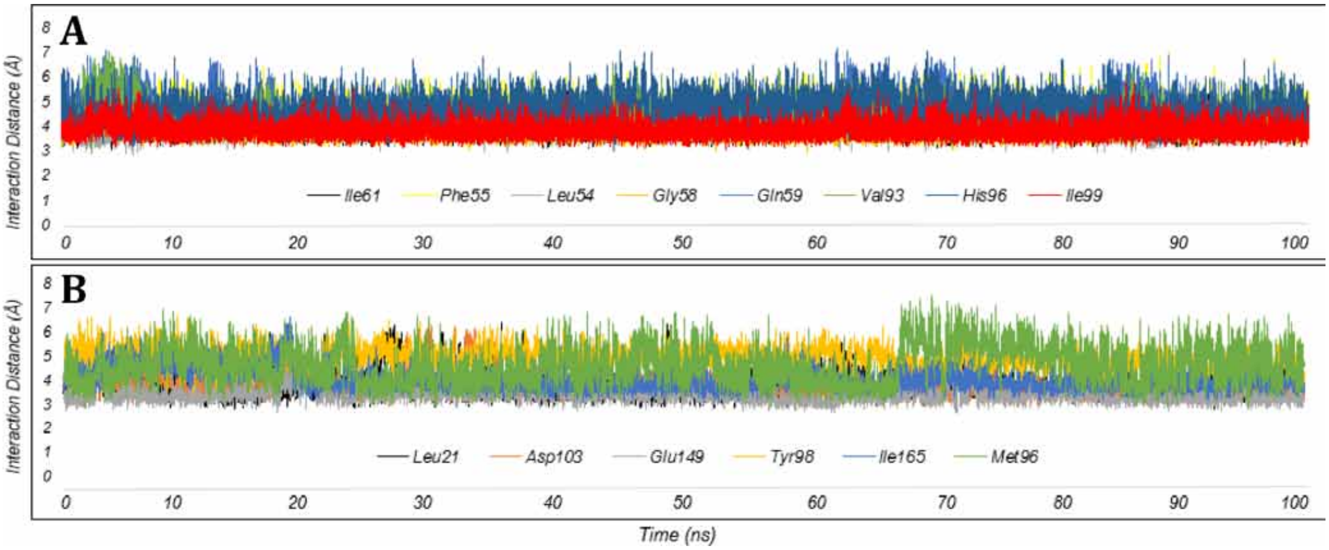

Supplementary Figure S4: Plot of Interatomic distances between relevant active site residues with (A) R-5s in MDM2 and (B) S-5s in IKKβ.
